# Takayasu's Arteritis presenting as a dissecting aortic aneurysm history: a case report

**DOI:** 10.1186/1757-1626-1-52

**Published:** 2008-07-21

**Authors:** Mortimer B O'Connor, Elizabeth Murphy, Neil O'Donovan, Michael Murphy, Mark J Phelan, Michael J Regan

**Affiliations:** 1 The Department of Medicine, South Infirmary – Victoria University Hospital, Cork, Ireland; 2 The Department of Radiology, South Infirmary – Victoria University Hospital, Cork, Ireland; 3 The Department of Rheumatology, South Infirmary – Victoria University Hospital, Cork, Ireland

## Abstract

**Introduction:**

Takayasu's Arteritis, formerly known as "pulseless disease", is a chronic idiopathic vasculitis which affects the large vessels in the body. First described in the 1800's, this rare condition is more commonly found in Asian women in their 40's. The aorta and its main branches are the primary vessels affected, with the most typical features reflected as ischemia or aneurysm formation. With Takayasu's Arteritis being a rare condition and its acute phase presentation often similar to other conditions, diagnosis is often difficult.

**Case Presentation:**

A 48 year old Irish Caucasian female, who presented as a typical history of an aortic dissection (chest pain radiating to her back in an interscapular region and a systolic blood pressure differential of 50 mmHg between her right and left upper limbs), was investigated with a number of imaging modalities and diagnosed with Takayasu's Arteritis, involving arteries affecting a number of organs. She was treated as per protocol for Takayasu's Arteritis. A diagnosis of cervical cancer quickly followed.

**Conclusion:**

This case report highlights that a differential diagnosis should never be dispelled based upon a "typical" history. The importance of modern day imaging techniques such as CT, MRI and angiography, can often be paramount to confirming a diagnosis and the extent of the pathology.

A possible link between Takayasu's Arteritis and gynaecological malignancies may exist.

## Introduction

Takayasu's arteritis (TA) is a chronic idiopathic vasculitis that variably involves the aorta and/or its main branches and the coronary and pulmonary arteries in 50–80% [1]. Inflammation results in stenosis, occlusion, or aneurysm formation [2]. Aneurysms may rarely progress to vascular rupture and death [3].

The first report of TA was by R Yamamoto in 1830, while the first presentation on TA was in 1905 by Mikito Takayasu at the 12th annual meeting of the Japan Ophthalmology Society, describing a patient with a peculiar optic fundus abnormality, characterised by coronal anastomoses [4]. K Ohnishi and T Kagoshima also presented similar cases and noted their patients lacked a palpable radial pulse [4]. The first autopsy on a patient with TA was carried out in 1940 by K Ohta [5].

The incidence of TA is about 2/10,000 person-years, with a ten fold predominance in women, especially under 40 years of age [6]. There is a marked ethnic preference [3], with high prevalence rates in Asian countries [7] and certain Central and South American countries [8], while less common in Caucasian populations [3].

Here we present a case of TA which presented with symptoms and signs of an aortic aneurysm. This case report highlights the importance of differential diagnoses and the importance of modern day imaging in the diagnosis. It is the first fully reported case, to our knowledge, with a full array of radiological investigation images confirming the extensive impact of TA on the vascular system.

## Case Presentation

A 48 year old Irish Caucasian female presented to our Emergency department with a history of central chest pain radiating to her back in an interscapular region. The pain was of sudden onset, with the chest pain resolving spontaneously but the back pain remaining. Of note the pain was associated with shortness of breath, nausea and sweating. Her background history included being a current chronic smoker (30 pack year history), epilepsy (since 16 years old), hypercholesterolemia, depression (post partners death from ischemic heart disease 18 months previous) and a strong family history of cardiac disease. Mediactions at presentation included: Dalamne 30 mg nocté, Efexor XL 150 mg PO BD and Tegretol 200 mg PO BD.

On examination she was noted to have a systolic blood pressure differential of 50 mmHg between her right and left upper limbs and had a weak radial pulse on the left side. The remainder of her examination was normal. All Cardiac bloods were within normal range.

A CT thorax and abdomen were carried out to investigate the principle differential diagnosis of a dissecting aortic aneurysm. This was ruled out and a stenosis in the left subclavian artery was noted. No bruit was audible over the subclavian artery. A differential diagnosis of TA recorded.

**Figure 1:**
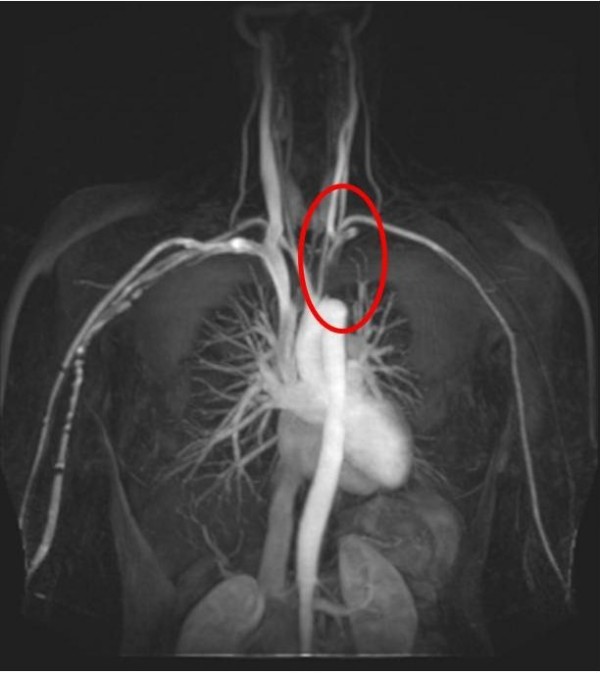
MRI showing complete occlusion of proximal 4 cm of left subclavian artery with retrograde filling via the left vertebral.

**Figure 2:**
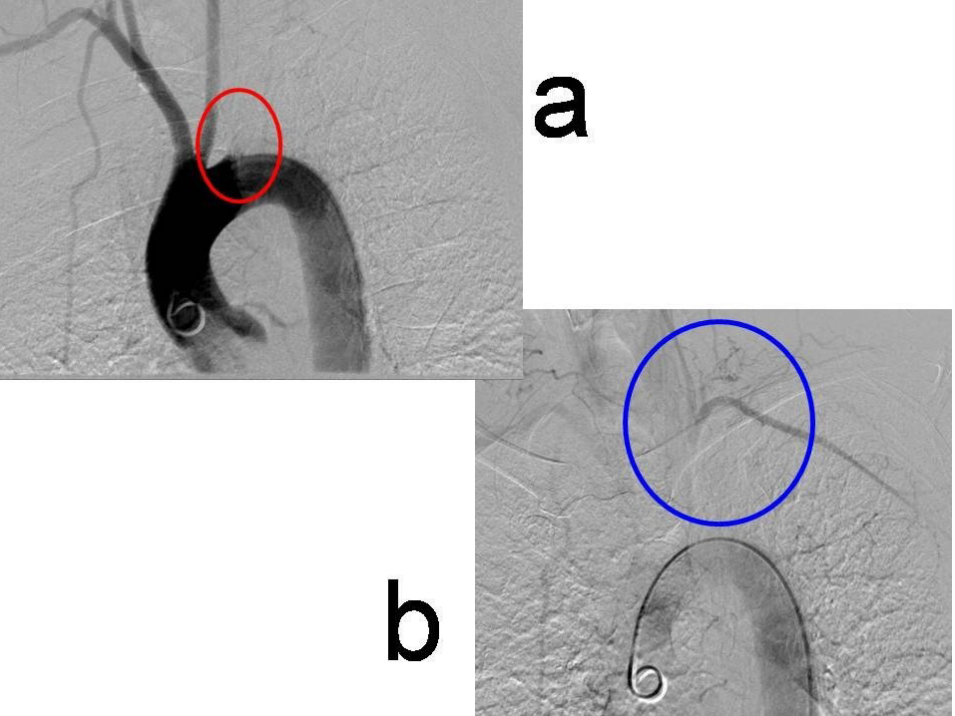
**Angiography of the great vessels**. 2a: showing a 4 cm occlusion of the of left subclavian artery (red circle) with 2b: demonstrating retrograde filling of the subclavian via the left vertebral (blue circle).

**Figure 3:**
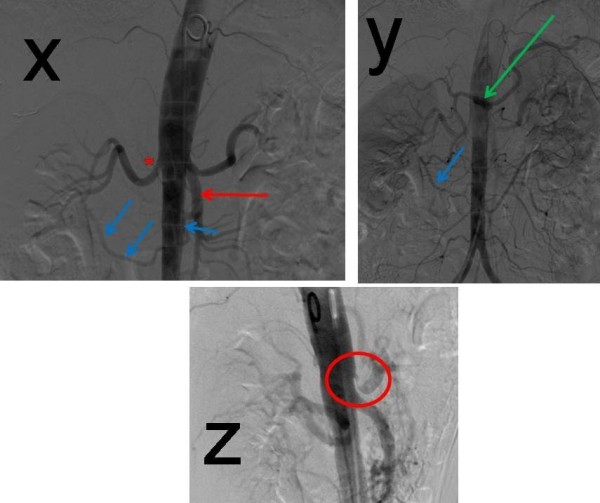
**3x, 3y: AP arteriography demonstrating retrograde filling of coeliac axis (green arrow) via a hypertrophied pancreatico-duodenal arcade (blue arrows) from the SMA (red arrow). **Additional mild right renal artery stenosis also noted (asterix). 3z: Lateral abdominal aortography demonstrating the tight stenosis of the coeliac origin (red circle).

Coronary angiography, MRI angiography (Figure 1), along with angiography of the great vessels/aorta (Figures 2a, 2b) were carried out and these confirmed the diagnosis of TA. The coronary angiogram showed an occluded PV branch of the left circumflex artery. The ateriogram showed a long segmental left subclavian artery stenosis and a significant stenosis in the origin of the celiac artery Figure 3.

A treatment regime of intravenous Methylprednisolone 1 g OD for three days followed by Prednisolone 60 mg PO OD for four weeks was commenced, along with Mycophenolate Mofetil 500 mg PO BD, Aspirin 75 mg PO OD, Clopidogrel 75 mg PO OD, Atorvastatin 20 mg nocté, nicotine replacement patch 14 mg TD OD and Fosavance 70 mg once weekly. These were well tolerated with no side effects experienced.

She was discharged, asymptomatic, 14 days post presentation, with an outpatients follow-up for four weeks. Advice regards cessation of smoking was given and adhered to, along with diet and lifestyle improvements. At her return outpatients appointment the Mycophenolate Mofetil was increased to 750 mg PO BD. The prednisolone was changed to 30 mg PO OD for two weeks followed by 25 mg × 1/52, 20 mg × 4/52, 10 mg × 4/52, 5 mg × 3/12 and then stopped.

It has been 10 months since this lady presented and currently she remains very well from a TA and cardiology viewpoint, however unfortunately she has since developed carcinoma of a gynaecological nature, cervical cancer. The cervical cancer diagnosis was confirmed two months after the diagnosis of TA. Regular follow-ups continue at increasing intervals from a TA perspective and she has been treated, successfully, for the malignancy.

## Discussion

Non-specific symptoms of inflammatory disease such as fever, night sweats, malaise and weight loss are common early in disease and often precede more specific features [2]. Arthralgia and myalgia are common. Some patients develop true arthritis or, less commonly, lupus-like rashes, erythema nodosum or glomerulonephritis.

With regards large vessel disease, the most typical features reflect ischaemia, or aneurysm formation in large vessels such as the aorta and its branches. "Aortic arch syndrome" is the term given to disease affecting the upper extremities, heart, neck and head. Patients often complain of arm claudication, and brachial and radial pulses are absent. Hence TA was previously called "pulseless disease"[4]. Blood pressure varies by more than 10 mmHg between the arms and a bruit may be audible over the subclavian artery. Aortic regurgitation, pulmonary hypertension, angina, congestive cardiac failure, vertigo, syncope, stroke and visual disturbance may occur. Descending aorta syndrome may cause renovascular hypertension, renal dysfunction, abdominal pain and acute abdominal bleeding or perforation of a viscus from infarction. Aortic aneurysms may develop and present with acute aortic dissection.

Confirmation of TA is best done by angiography or MRI angiography. The most common lesion is a smooth, concentric, arterial or aortic narrowing (85%). Irregular narrowing, complete occlusion and fusiform or saccular aneurysms are less commonly seen. Changes may be focal or segmental and are distinguished from arteriosclerosis and fibromuscular dysplasia. Contrast-enhanced magnetic resonance perfusion imaging, ultrasonography and positron emission tomography are new, non-invasive methods of assessment that are likely to replace conventional angiography.

In the early phase, features of inflammation are present clinically and on blood tests (acute phase response). However, the disease may not present until after arterial damage has occurred. At this stage, there may be little evidence of current inflammation.

Since the aetiology of TA is still not fully understood, treatment is not curative, but rather primarily medical and symptomatic. Renovascular hypertension, coarctation of aorta, severe cerebral ischaemia, and severe aortic regurgitation causing congestive heart failure, or progressive aneurismal enlargement or dissection may all require prompt surgical treatment.

Surgery, angioplasty or stenting [2] is only required in a minority of patients and should be postponed until the inflammatory component of the disease has been controlled if possible. Regards medical management of TA, it is treated initially as giant cell arteritis (GCA) would be, with high-dose prednisolone, 40–60 mg per day, as soon as the diagnosis is suspected. Patients with the HLA A24-B52-DR2 haplotype may require larger doses of corticosteroids for longer periods than patients without this haplotype [9]. TA response is often slower than that seen in GCA and cytotoxic drugs are more likely to be needed to reduce the toxicity associated with high dose corticosteroids [10]. If the patient presents after arterial damage has occurred, the disease is less likely to respond to immunosuppression. There is an increased risk of premature atherosclerosis and patients should be screened for other cardiovascular risk factors.

The risk of malignancy associated with TA has been reported in the literature. Salman et al have reported Meig's Syndrome in a patient with TA in 2005 [11], while others have reported associations with Epstein Barr Virus [12] and Leukemias [13,14]. The association of malignancy with TA is currently unclear as only case reports have been published. From the case presentation we know that this lady was diagnosed with cervical cancer. This diagnosis was made on colposcopy approximately two months after her TA diagnosis. The link between TA and cervical cancer cannot be confirmed based upon this case report, but it does suggest a possible association. Further reports or a case series along with molecular associations would be required to strengthen a causative link between TA and cervical cancer. This is the first case report, known to the authors, of TA with a subsequent, possibly related, diagnosis of cervical cancer. Therefore the possibility of monitoring for malignancy in the setting of a TA diagnosis may be beneficial.

## Conclusion

TA can present in wide variety of ways, many with a typical history of other conditions. The use of steroids is paramount to the acute medical treatment but not curative. Surgery, angioplasty or stenting is only required in a minority of patients.

For a definitive diagnosis the use of modern day imaging such as CT, MRI and angiography is vital.

From this case report we learn the importance of keeping an open mind with differential diagnoses, despite a "typical" presenting history, and the need for confirmatory investigations and therefore appropriate treatment.

The possible link between malignancy, especially gynaecological malignancies, and TA needs to be explored more extensively in future studies.

## Abbreviations

TA: Takayasu's arteritisl; CT scan: Computerised Tomography scan; MRI scan: Magnetic resonance imaging scan; PV: posterior ventricle; GCA: giant cell arteritis; HLA: Human leucocyte antigen.

## Competing interests

The authors declare that they have no competing interests.

## Authors' contributions

MOC interviewed the patient, reviewed the radiological images and was the major contributor in writing the manuscript. EM interviewed the patient. NOD and MM were the principle reviewers of the radiological images. MP contributed in writing the manuscript. MR interviewed the patient and contributed in writing the manuscript. All authors read and approved the final manuscript.

## Consent

Written informed consent was obtained from the patient for publication of this case report and accompanying images. A copy of the written consent is available for review by the Editor-in-Chief of this journal.
